# Hotspots of single-strand DNA “breakome” are enriched at transcriptional start sites of genes

**DOI:** 10.3389/fmolb.2022.895795

**Published:** 2022-08-15

**Authors:** Huifen Cao, Yufei Zhang, Ye Cai, Lu Tang, Fan Gao, Dongyang Xu, Philipp Kapranov

**Affiliations:** Institute of Genomics, School of Medicine, Huaqiao University, Xiamen, China

**Keywords:** DNA damage, breakome, single-strand breaks, DNA break hotspot, transcription start site, promoter, cap analysis of gene expression

## Abstract

Single-strand breaks (SSBs) represent one of the most common types of DNA damage, yet not much is known about the genome landscapes of this type of DNA lesions in mammalian cells. Here, we found that SSBs are more likely to occur in certain positions of the human genome—SSB hotspots—in different cells of the same cell type and in different cell types. We hypothesize that the hotspots are likely to represent biologically relevant breaks. Furthermore, we found that the hotspots had a prominent tendency to be enriched in the immediate vicinity of transcriptional start sites (TSSs). We show that these hotspots are not likely to represent technical artifacts or be caused by common mechanisms previously found to cause DNA cleavage at promoters, such as apoptotic DNA fragmentation or topoisomerase type II (TOP2) activity. Therefore, such TSS-associated hotspots could potentially be generated using a novel mechanism that could involve preferential cleavage at cytosines, and their existence is consistent with recent studies suggesting a complex relationship between DNA damage and regulation of gene expression.

## Introduction

Genome within each living cell is constantly subjected to exogenous and endogenous assaults that can result in a multitude of different changes to DNA structure, including various chemical modifications of DNA as well as physical breaks in DNA chains, among others (Jackson and Bartek, 2009). These changes, collectively referred to as DNA damage, can have a variety of broadly recognized detrimental effects on the organism, such as, if not properly repaired, permanent DNA mutations potentially leading to cancer, cell death, or cell depletion and premature aging (Hoeijmakers, 2009; Maynard et al., 2015; Ou and Schumacher, 2018). A discontinuity located on just one strand of a DNA double-helix, or SSB, represents one of the most common types of DNA damage (Caldecott, 2008). Such breaks can be caused by oxidative damage or occur as intermediates of normal cellular processes, for example, SSBs are induced by topoisomerases in order to change the topology of DNA or, they can also occur during the repair of other types of DNA lesions (Caldecott, 2008; Reynolds and Stewart, 2013). Persistent SSBs can have a variety of detrimental effects on the cell: they can be converted into highly toxic double-strand breaks (DSBs) and collapse DNA replication forks (Kuzminov, 2001), inhibit the progression of RNA polymerase (Kathe et al., 2004), potentially cause DNA sequence changes (Cao et al., 2019) or induce apoptosis (Ljungman and Zhang, 1996; Ljungman et al., 1999). The importance of proper repair of this type of lesion is underscored by the presence of a dedicated single-strand break repair (SSBR) system, defects in which can lead to sensitivity to genotoxic stress, embryonic lethality, and neurodegenerative diseases (Caldecott, 2008; Reynolds and Stewart, 2013; Rulten and Caldecott, 2013).

Despite the importance of SSBs, the patterns of their distribution genome-wide are still not well understood. In fact, the methods to map these lesions genome-wide and with nucleotide level resolution, such as SSiNGLe (Cao et al., 2019), GLOE-seq (Sriramachandran et al., 2020), Nick-seq (Cao et al., 2020), and DENT-seq (Elacqua et al., 2021), have been developed only very recently and rely on different molecular strategies to detect SSBs. SSiNGLe and GLOE-seq directly tag 3′-OH termini of DNA breaks either by addition of polyA tails mediated by terminal transferase (TdT) (Cao et al., 2019) or by ligation to a double-strand adaptor (Sriramachandran et al., 2020). Nick-seq and DENT-seq on the other hand rely at least in part on nick-translation primed by the 3′-OH termini of SSBs. Nick-seq is based on an elaborate procedure where each SSB has to be detected by a combination of two approaches, nick-translation and TdT-mediated tailing (Cao et al., 2020), whereas DENT-seq relies on nick-translation in presence of degenerate nucleotides to generate specific mutation spectra adjacent to the SSB (Elacqua et al., 2021). So far, however, only SSiNGLe and GLOE-seq have been applied to generate profiles of endogenous mammalian SSBs and these efforts have been limited to very few cell types (Cao et al., 2019; Sriramachandran et al., 2020). Thus, our understanding of mammalian SSB “breakome” is still at its very beginning.

**FIGURE 1:**
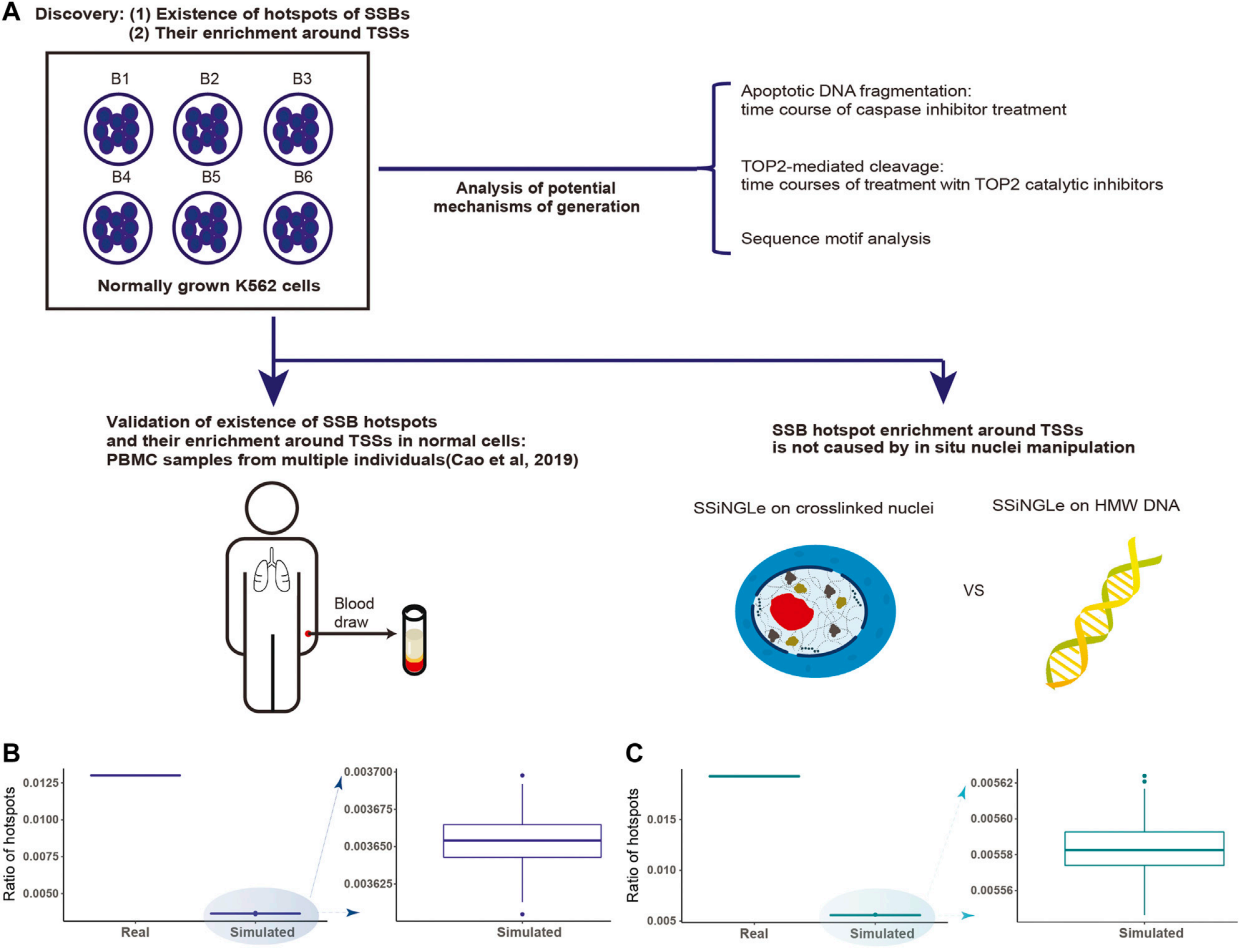
Discovery and analysis of SSB hotspots. **(A)** Schematics illustrating the discovery and characterization of SSB hotspots performed in this work. A hotspot had to be found in at least 2 independent biological samples represented either by different batches of cells (B1-B6 for K562) or PBMCs extracted from 66 individuals. **(B,C)** Discovery of SSB hotspots in K562 and PBMCs. The fractions of hotspots in the genome (Y-axes) for the real and simulated data (X-axes) from **(B)** K562 and **(C)** PBMCs are shown.

Therefore, in this work, we further explored the human SSB “breakome” using one of the above methods, SSiNGLe, developed by our group (Cao et al., 2019) with specific emphasis on breaks that can be consistently found in the same genomic positions in different cells of the same or different human cell types as illustrated in Figure 1A. Theoretically, such positions representing hotspots of SSBs would more likely correspond to physiologically relevant breaks. Interestingly, we found that such SSB hotspots are enriched around TSSs of genes, and this enrichment can be found in both cancerous and normal human cell types. We further show that the enrichment is not an artifact of formaldehyde crosslinking or micrococcal nuclease (MNase) fragmentation of DNA in crosslinked nuclei used in the standard SSiNGLe technique (Cao et al., 2019) by performing “breakome” profiling directly on high-molecular-weight (HMW) DNA. Finally, we show that the hotspots of breaks enriched around the TSSs could be generated by a novel mechanism, and discuss the potential mode of their generation and the implications of their existence for the regulation of gene expression.

## Materials and methods

### Biological material

Human CML leukemia cell line K562 was obtained from the Cell Bank of the Chinese Academy of Sciences. Cells were cultured in RPMI 1640 (Thermo Fisher Scientific) supplemented with 10% (v/v) heat-inactivated fetal bovine serum (FBS, ExCell Bio) and 1% (v/v) pen-strep (Thermo Fisher Scientific) at 37°C and 5% CO_2_.

### SSB profiling

Three million K562 cells were seeded at 1 million cells per ml of medium per well in 6-well plates. After 16 h, the cells were separately treated with 0.1% DMSO, 20 μM Z-DEVD-FMK (AbMole), 100 μM ICRF-187 (Selleck), and 100 μM merbarone (Merck) and incubated for 6, 12, 24, 36, or 48 h. All drugs were dissolved in DMSO, the concentration of which was kept at 0.1% in all treatments. Each treatment was done in two independent biological replicas. The cells were used as input in the standard SSiNGLe-ILM protocol on crosslinked nuclei (https://protocolexchange.researchsquare.com/article/pex-920/v2).

For SSiNGLe on HMW DNA, the DNA was extracted directly from K562 cells using the TIANamp Genomic DNA Kit (TIANGEN Biotech, DP304) according to the manufacturer’s protocol. One hundred nanograms of the DNA were used directly as input into the SSiNGLe-ILM protocol (https://protocolexchange.researchsquare.com/article/pex-920/v2) at the polyA-tailing step with only one modification at the Illumina library construction stage: Step 19 in the procedure corresponding to the 2nd round PCR was performed with all (instead of just the 4 µl used in the standard protocol) DNA from the 1st round PCR. The latter was purified with the 2x volume of the VAHTS DNA Clean Beads (Vazyme) before adding to the 2nd round PCR. Sequencing for all SSB profiling experiments was performed on the Illumina NovaSeq platform using paired-end 150 bp strategy at 1-GB (gigabase) scale by Novogene Corporation (Beijing).

### SSB mapping

Assigning SSBs to genomic positions for the data generated in this work was performed the same way as in the SSiNGLe-ILM protocol (https://protocolexchange.researchsquare.com/article/pex-920/v2) with the exception that an additional filter was used: a read-pair was used only if the first base of the read 2 aligned to the genome. The coordinates of SSBs from PBMCs were derived from our previous study (Cao et al., 2019). Genomic position of a break between bases N and M in the sequence 5′-NM-3′ was assigned to the base N. All analyses were performed using unique genomic positions of SSBs, i.e., for each biological replica, a genomic position was counted only once irrespective of the number of SSBs detected there. Only positions mapping outside of the repeated regions as defined by the RepeatMasker track (Jurka, 2000) of the UCSC Genome Browser were used (Kent et al., 2002). The simulation of the SSB hotspot proportion expected by chance was performed 100 times by the “sample” function in the R environment, and the corresponding *p*-values were calculated by the two-sided Student t-test. Based on the outcome of the simulations, the hotspots of SSBs were defined as the positions shared by at least 2 biological replicas in each cell type.

### Definition of the TSSs

BED files containing positions of CAGE tags generated using Helicos sequencing platform by the FANTOM5 consortium (Lizio et al., 2014) for untreated K562 cells (FANTOM Source Names 10454-106G4, 10824-111C5, 10824-111C6, and 10824-111C7) and normal peripheral blood mononuclear cells (PBMCs; FANTOM Source Names 11231-116C7, 11231-117C7, and 11231-118C7) were downloaded from https://fantom.gsc.riken.jp/5/datafiles/latest/basic/. The CAGE tags from the multiple BED files for the sample cell type were combined for the downstream analysis. The coordinates of the annotated TSSs were derived from the UCSC Genes genome annotation database (Hsu et al., 2006) downloaded from the UCSC Genome Browser (Kent et al., 2002).

For each cell type, CAGE tags found by at least 2 sequencing reads (i.e. having depth ≥2) were defined as the CAGE peaks. Then, for each annotated gene expressed in either K562 or PBMC, the actual TSS used for all downstream analyses was defined as the CAGE peak with the maximum depth of all CAGE peaks mapping within ±200 bp of the annotated TSS. Using this approach, 9,732 and 10,901 genes were found to be expressed in K562 and PBMCs and could be assigned to a TSS based on a CAGE peak. The overlaps among the TSSs, CAGE peaks, and SSBs were performed in a strand-specific fashion with respect to the template or non-template strand of the transcript represented by the TSS or CAGE peak: an SSB mapping to either the opposite or same strand of a TSS or CAGE peak represented the template or non-template strand match respectively.

### Aggregate plots and TSS-SSB enrichment ratios

For each SSB or hotspot position, the distance to the TSSs was calculated, and only the absolute shortest distance was kept. SSBs or hotspots mapping upstream or downstream of the corresponding genes were then assigned negative or positive distances respectively. The ± 5,000 bp region around each TSS was split into 500 non-overlapping 20-bp bins. The fraction of non-repeat sequence in each bin around each TSS was calculated and then used to calculate the average non-repeat ratio of each bin around all TSSs. For each sample *i*, the normalized density D_*ij*_ of SSBs or hotspots in each bin *j* (1-500) across all expressed genes in the sample was defined using the following two formulas:

$$NR_{j}=\left(\sum_{\mathrm{k=}1}^{\mathrm{k=n}}\left(\frac{\mathrm{LN}R_{\mathrm{kj}}}{\mathrm{20}}\right)\right)/n,$$

$$D_{\mathrm{ij}}=\frac{N_{\mathrm{ij}}^{*}10^{6}}{NR_{j}^{*}T_{i}},$$

where NR_*j*_ is the average non-repeat ratio for the bin *j* across all expressed genes assigned to a TSS, *n* is the number of expressed genes (9,732 and 10,901 for respectively K562 and PBMCs, see above); LNR_*kj*_ is the total length of the non-repeat region in the bin *j* in gene *k* and the LNR_*kj*_/20 is the non-repeat ratio of that bin, N_*ij*_ is the total number of the corresponding positions of SSBs or hotspots mapping to the bin *j* in the sample *i*, and T_*i*_ is the total number of the corresponding positions in the sample.

**FIGURE 2:**
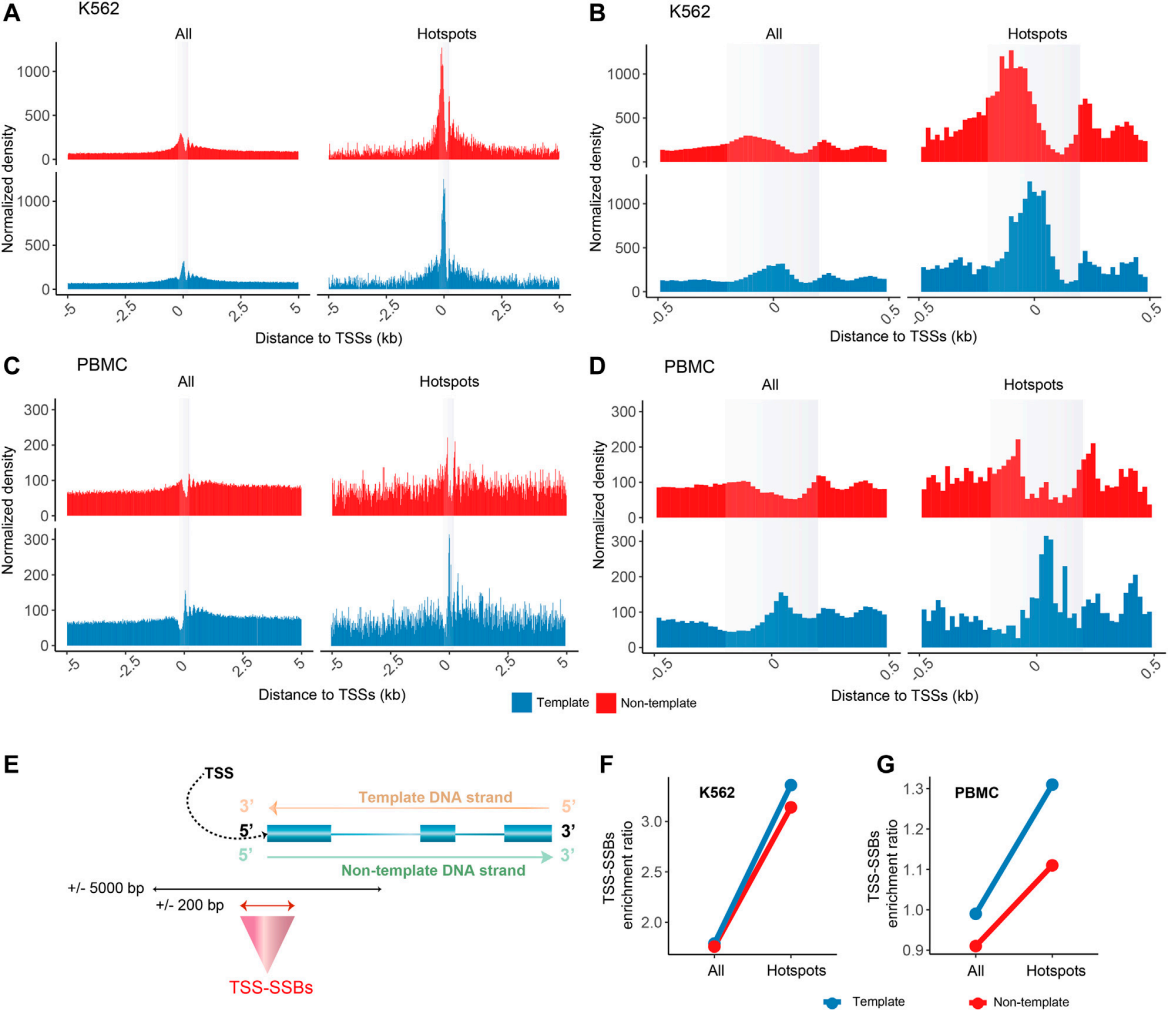
Enrichment of SSB hotspots around TSSs. Aggregate plots of the normalized densities (Y-axes) of the positions of all SSBs or hotspots within ±5,000 bp **(A,C)** or ±500 bp **(B,D)** of the TSSs for K562 **(A–B)** and PBMCs **(C–D)**. **(A–D)** The opaque vertical rectangles represent the ±200 bp areas around the TSSs. The aggregate plots for the template and non-template strands of genes are shown separately as illustrated in **(E)**. **(F–G)** The TSS-SSBs enrichment ratios (Y-axes) for all breaks and hotspots on either template or non-template strands for K562 **(F)** and PBMC **(G)**.

**FIGURE 3:**
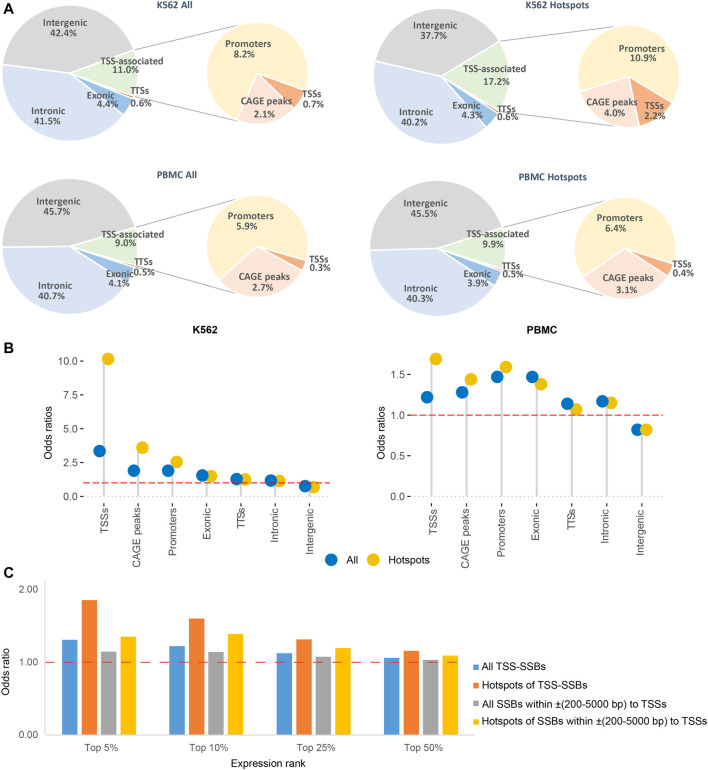
Distribution of all breaks and hotspots near TSSs and elsewhere in the genome. **(A)** Fractions of all breaks and hotspots mapping to within ±200 bp of TSSs, CAGE peaks or TTSs and inside promoters, exonic, intronic, or intergenic regions as described in Materials and Methods for K562 (top) and PBMC (bottom). **(B)** Odds ratios (Y-axes) of enrichment of all breaks and hotspots in the different types of genomic elements (X-axes). The red dashed horizontal lines represent odds ratios of 1 corresponding to no enrichment. See Supplementary Table S3 for the exact numbers and the corresponding *p*-values, and Materials and Methods for more details. **(C)** The odds ratios of enrichment (Y-axes) of all SSBs or hotspots within ±200 bp or ±(200–5,000) bp of the TSSs for top 5%, 10%, 25% and 50% expressed genes compared to the randomly simulated data (Materials and Methods).

The enrichment of SSBs or hotspots in the immediate vicinity (±200 bp) around the TSSs shown in Figures 2F, G; Figure 3C and Figure 5 was calculated for each sample *i* relative to the background defined as ±5,000 bp around the TSSs as the TSS-SSBs enrichment ratio R_*i*_ using the following formula:

$$R_{i}=\frac{M_{i}^{\mathrm{200}}/M_{i}^{\mathrm{5000}}}{L_{i}^{\mathrm{200}}/L_{i}^{\mathrm{5000}}},$$

where $M_{i}^{200}$ and $M_{i}^{5000}$ are respectively the total numbers of positions of SSBs or hotspots within ±200 bp or ±5,000 bp of TSSs in the sample; and $L_{i}^{\mathrm{200}}$ and $L_{i}^{\mathrm{5000}}$ are respectively the total lengths of the non-repeated sequences within ±200 bp and ±5,000 bp around the TSSs. Note that $L_{i}^{200}$ and $L_{i}^{5000}$ would be the same for each sample of the same cell type, but differ between K562 and PBMC because each cell type has a different set of TSSs. The *p*-value of the enrichment ratio was calculated by the two-sided binomial test.

### Association of SSBs or hotspots with TSSs

For each cell type, we calculated the number of SSBs or hotspots located in each of the following 7 types of genomic elements: 1) within ±200 bp of the TSSs; 2) within ±200 bp of any CAGE peak; 3) mapping to promoters defined by chromatin state analysis by the ENCODE/Broad consortium (Ernst et al., 2011); 4) within ±200 bp of the transcription termination sites (TTSs) defined by the 3′ end coordinates of the longest transcript of each gene; 5) exonic regions; 6) intronic regions, and 7) intergenic regions. TTSs, exonic and intronic regions, were defined based on the longest transcript of each gene from all annotated human genes irrespective of the expression status. Every SSB or hotspot could be assigned to only one element type by a hierarchical strategy, so that breaks assigned to element type 1 cannot be assigned to element types 2-7, and breaks assigned to types 1 or 2 cannot be assigned to the types 3-7 and so on. The promoters from the 7 human cell lines were downloaded from the “Chromatin State Segmentation by HMM from ENCODE/Broad” track of the UCSC genome browser (Kent et al., 2002) and the promoters from all 7 cell lines and the 3 categories (“Active”, “Weak”, and “Poised”) were merged. The enrichment of the overlaps relative to the random chance was calculated as the odds ratio OR_*i*_ for each cell type and each of the 7 types of genomic elements *i* as

$$OR_{i}=\frac{M_{i}/T_{i}}{L_{i}/\mathrm{LG}},$$

where M_*i*_ is the number of positions of SSBs or hotspots mapping to the element type *i* in a given cell type; T_*i*_ is the total number of positions of SSBs or hotspots in a given cell type; L_*i*_ is the total non-repeat length of the genomic element and LG is the total non-repeat length of the genome. For this analysis, SSBs were defined as any SSB found in at least one of the six bio-replicas of normally grown K562 cells or one of the 66 PBMC samples. As stated above, the hotspots were defined as SSBs found in at least 2 bio-replicas of K562 cells or 2 PBMC samples. The *p*-values of the overlaps were calculated by a two-sided binomial test in the R environment.

### TSS-SSBs vs. gene expression analysis

K562 were grown under the conditions described above without any drug treatments. The protocol for total RNA extraction was the same in Cao et al. (2021). The total RNA samples were used for RNA-seq library construction using rRNA depletion strategy such that both polyA+ and polyA−transcripts are included. The library construction and the Illumina sequencing using a paired-end 150 bp strategy on 10-GB scale were outsourced to Novogene Corporation (Beijing). Only read pairs where each read was ≥30 bases after adaptor trimming and each base had the Phred quality score ≥20 were selected. The read pairs were aligned to the GRCh37/hg19 assembly of the human genome by the Tophat software (Trapnell et al., 2009), and the uniquely mapping read pairs were used to calculate the FPKM for each transcript. For this purpose, we ignored the exon-intron structures of genes and only used the start and end coordinates and strand information as the input of Tophat and Cufflinks software with the default parameters (Trapnell et al., 2010). Therefore, the resulting FPKM values represent both exonic and intronic signals, and only the genes with TSSs (CAGE tags) were kept for downstream analysis.

The genes were ranked by the average FPKM values of two biological replicas of the untreated K562 samples. Random positions of 11,687,672 breaks were generated using R (version 4.1.0), assigned to the coordinates in the non-repeat genomic regions by BEDOPS (version 2.4.40) (Neph et al., 2012) and then mapped to genomic regions located within ±200 bp and ±(200–5,000) bp of the TSSs of the top 5%, 10%, 25% and 50% highly expressed genes to generate the expected fractions of breaks mapping to each distance range of each expression bin. The observed fractions of SSBs and hotspots located within ±200 bp or ±(200–5,000) bp of the TSSs for the genes in each expression bin were then calculated based on the actual data, and the odds ratios were then defined as the observed fractions of all SSBs or hotspots divided by the corresponding expected fractions (Figure 3C). The *p*-values of different comparisons were obtained by a one-sided Student’s *t* test (Supplementary Table S4).

## Results

### Hotspots of human SSBs exist and are enriched in immediate proximity to TSSs

In our previous study of genome-wide distribution of SSBs in mammalian cells using the SSiNGLe method, we found a very high complexity of breaks with most of them represented by single occurrences (Cao et al., 2019). These results were consistent with the previous knowledge that SSBs represent one of the most common DNA lesions with an estimated as many as 55,000 breaks per mammalian cell (Tice and Setlow, 1985) and outnumber DSBs by three orders of magnitude (Caldecott, 2008). Many of these breaks likely represent random, background events that occur in different genomic locations in different cells. However, it is plausible that certain genomic positions are favored to have SSBs in different cells of a specific cell type, due to either lower rates of repair at those positions or higher occurrence of cleavage by either exogenous or endogenous factors. Breaks at such locations could have more pronounced biological effects than breaks occurring randomly in the genome.

Therefore, as the first step, we tested whether positions, where SSBs tend to occur more often in different cells, exist in the human genome. To do so, we have performed SSB profiling using SSiNGLe on 6 batches of independently grown human leukemia K562 cells. We could identify 150,052 unique single-nucleotide positions in the human genome where breaks occurred in at least 2 cell batches that represented 1.3% (150,052 of 11,532,044) of all unique positions found in at least one batch of cells (Supplementary Table S1). To test whether the positions of SSBs shared by different batches represented random occurrence or true hotspots of SSBs, we performed 100 simulations with random data containing the same numbers of samples and breaks per sample. The number of shared positions was always significantly higher (*p*-value < 2.16E-16, two-sided Student t-test) in the real data compared to the simulated one (Figure 1B, Materials and Methods, Supplementary Table S1), strongly suggesting that the shared positions represent true hotspots of SSBs. We could also identify hotspots shared by at least 3, 4, 5, and 6 cell batches, however, their numbers were significantly smaller: 4,620, 617, 240 and 99 respectively (Supplementary Table S1). Nonetheless, the observed numbers of such hotspots were also significantly higher than expected by chance (all *p*-values < 2.16E-16, two-sided Student t-test).

We then tested whether the existence of hotspots was a unique property of a cancerous state. To address this, we took advantage of the SSB profiles of normal human PBMC samples isolated from 66 individuals and generated by us using SSiNGLe in the previous study (Cao et al., 2019). We could identify 337,664 hotspots present in at least two individuals that represented 1.93% of all positions found in at least one sample (Supplementary Table S1). And, similar to the results with K562, the occurrence of the hotspots was statistically significant (*p*-value < 2.16E-16, two-sided Student t-test) using the simulation analysis (Figure 1C, Supplementary Table S1). As in the K562, we could also identify hotspots shared by at least 3, 4, and more individuals (Supplementary Table S1). However, while higher than expected by chance, the observed numbers of such hotspots were significantly smaller than those found in at least 2 individuals (Supplementary Table S1). Therefore, to ensure comprehensive coverage, for all subsequent analyses, we will define SSB hotspots as unique genomic positions of breaks found in at least 2 independent biological samples of the same cell type. Strikingly, such hotspots had a tendency to overlap between different cell types. Of the 150,052 and 337,664 hotspots found in K562 and PBMC, 1,180 were in common. This overlap was highly significant as represented by the odds ratio of 38.41-fold over what would be expected by random chance and the *p*-value < 2.16E-16 (two-sided binomial test).

As shown in Figure 2, we discovered that the SSB hotspots had a striking tendency to be enriched around TSSs compared with all SSBs, the vast majority of which represented SSBs found in just one sample as mentioned above. Since TSSs annotated in genomic databases do not always represent the actual TSSs used in a particular cell type and not all genes are expressed in a given cell type, we defined the most abundant TSS for each expressed gene based on the FANTOM5 CAGE tags that mark 5’ positions of capped transcripts (Lizio et al., 2014) that were obtained from K562 cells or PBMCs (Materials and Methods). We then calculated cumulative distributions of all SSBs or just the hotspots within ±5,000 bp of the TSSs for the 9,732 and 10,901 genes found to be expressed in K562 and PBMCs (Materials and Methods). In this analysis, we treated breaks occurring on the template and non-template strands of these genes separately.

Strikingly, the SSB hotspots had a prominent enrichment in the immediate vicinity (±200 bp) of TSSs on both strands as shown on the aggregate plots in Figures 2A–D. This enrichment was most pronounced in the K562 cells (Figures 2A, B), but was also apparent in the PBMCs (Figures 2C,D). Therefore, we named the breaks or hotspots found within ± 200 bp of TSSs as TSS-SSBs (Figure 2E). To further quantify the enrichment of TSS-SSBs relative to the background (defined as ±5,000 bp of TSSs), we calculated the TSS-SSBs enrichment ratio for all breaks and hotspots in each cell type (Figure 2E, Materials and Methods). As shown in Figures 2F,G, the corresponding ratios for all breaks on the template and non-template strands were 1.79 and 1.76 in K562, and 0.99 and 0.91 in PBMCs (Supplementary Table S2). However, the corresponding ratios increased to 3.36 and 3.14 in K562 and 1.31 and 1.11 in PBMCs when only the hotspots were considered (Supplementary Table S2). While in K562 hotspots on both template and non-template strands were prominently enriched, even though the former was a bit higher than the latter (Figures 2A,B and F), in PBMCs the enrichment was clearly most prominent on the template strand (Figures 2C,D,G).

The observed enrichment pattern in the aggregate plots was not a product of a very high number of breaks around TSSs of only a few genes. Overall, 2,147 and 1,184 genes contained hotspots of breaks in either strand within ±200 bp of TSSs in K562 or PBMCs respectively. Of those, 70.3% (1,510 of 2,147) and 88.9% (1,053 of 1,184) contained only one hotspot in the respective cell types, 18.6% (399 of 2,147) and 9.5% (112 of 1,184) contained two hotspots, and only 2% and 0.6% genes contained more than five hotspots. The observed enrichment could also not be explained by PCR duplicates since all analyses in this work were done on unique genomic positions.

Even though TSS-SSBs represented a minority (respectively 0.7% and 2.2%) of all breaks or hotspots found anywhere in the genome in K562 (Figure 3A), their associations with TSSs were highly significant: the corresponding odds ratios for all breaks and hotspots were 3.4 and 10.2 with the respective *p*-values < 2.16E-16 (two-sided binomial test, Figure 3B, Supplementary Table S3, Materials and Methods). The corresponding values for the PBMCs were lower, yet still statistically significant, for example, the corresponding odds ratios for all breaks and hotspots were 1.2 and 1.7, and the respective *p*-values < 2.16E-16 (two-sided binomial test) in this cell type (Figure 3B, Supplementary Table S3, Materials and Methods).

In the above analyses, we used only one TSS per annotated gene. However, genes are known to have multiple TSSs (Denoeud et al., 2007) and the human genome also encodes multiple unannotated transcripts (Kapranov and St Laurent, 2012; St Laurent et al., 2015). Thus, the number of hotspots associated with TSSs mentioned above most certainly under-counted the total number of breaks associate with TSSs. To generate a more comprehensive estimate of these values, we calculated the numbers of all breaks and hotspots associated with all other CAGE peaks found in K562 and PBMC (Materials and Methods). Also, since it is possible that some active TSSs could be missed by the CAGE analysis, we estimated the numbers of additional breaks and hotspots mapping to promoters annotated by the ENCODE/Broad consortium (Materials and Methods, (Ernst et al., 2011)).

All breaks and hotspots have shown statistically significant associations with the TSSs, CAGE peaks, and promoters as evidenced by all odds ratios being >1 in each cell type (Figure 3B, see Supplementary Table S3 for the exact values for the odds ratios and *p*-values). Also, as shown in Figure 3B, in each TSS-associated comparison, the odds ratios for the hotspots were higher than those for all breaks, further supporting increased associations between the TSSs and hotspots of breaks. Altogether, respectively 11 and 17.2% of all breaks or hotspots could be associated with TSSs, GACE peaks or promoters in K562, and respectively 9% and 9.9% in PBMCs (Figure 3A). Interestingly, the other breaks and hotspots have shown statistically-significant associations with exons, introns, and TTSs of genes (odds ratios >1), and depletion in the intergenic regions (Figure 3B, Supplementary Table S3).

Still, among all tested genomic elements, SSB hotspots have shown the strongest enrichment in the immediate vicinity of TSSs, which was especially apparent in K562 cells (Figure 3B, Supplementary Table S3). This prompted us to investigate a possible connection between the presence of SSBs near TSSs and levels of gene expression in this cell line. To do so, we ranked genes based on expression in normally grown K562 cells estimated using RNA-seq analysis (Materials and Methods). We then calculated the odds ratios of enrichment of either all SSBs or hotspots within two distance bins, ±200 bp and ±(200–5,000) bp, around TSSs of the top 5%, 10%, 25% and 50% expressed genes. We found that, in general, the presence of either all breaks or hotspots within both distance bins was significantly associated with higher expression, as evidenced by the increase in the corresponding odds ratios with the expression levels (Figure 3C, Supplementary Table S4). Genes associated with hotspots had a significant tendency to have higher expression than those associated with all breaks in both distance bins: *p*-values of 0.03 and 0.02 (one-sided Student t-test) for the respectively ±200 bp and ±(200-5,000) bp bins (Figure 3C, Supplementary Table S4). Finally, all breaks and hotspots found in the proximal distance bin (±200 bp) had higher expression than those in the ±(200-5,000) bp bin with the corresponding *p*-values of 0.04 and 0.05 (one-sided Student t-test) (Figure 3C, Supplementary Table S4).

### Hotspot enrichment around TSS is not an artifact of *in situ* nuclei manipulation

**FIGURE 4:**
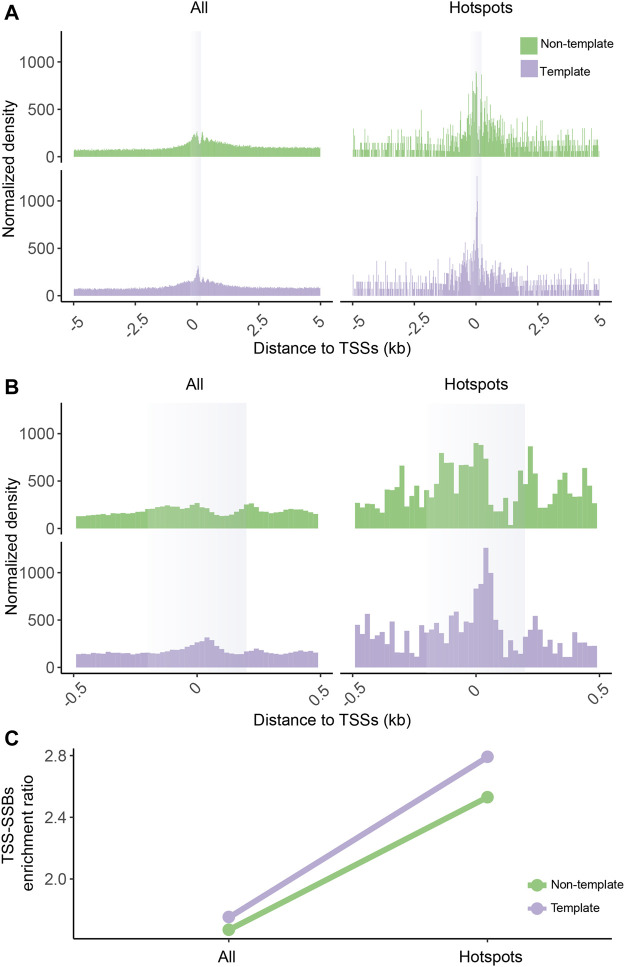
Enrichment of SSB hotspots around TSSs in SSiNGLe performed on HMW genomic DNA isolated from K562. Aggregate plots of the normalized densities (Y-axes) of the positions of all SSBs or hotspots on the template or non-template strands within ±5,000 bp **(A)** or ±500 bp **(B)** of the TSSs. **(A–B)** The opaque vertical rectangles represent the ±200 bp areas around the TSSs. **(C)** The TSS-SSBs enrichment ratios (Y-axes) for all breaks and hotspots on either template or non-template strands.

We further tested whether the enrichment of the hotspots around TSSs could be an artifact of formaldehyde crosslinking and/or MNase fragmentation performed on crosslinked nuclei as part of the standard SSiNGLe protocol to prevent mechanical breaks caused by shearing of HMW genomic DNA during purification (Cao et al., 2019). For example, it is conceivable that breaks in promoters of transcribed genes are better detectable by SSiNGLe due to better accessibility to MNase in crosslinked nuclei or that MNase has sequence preferences. Furthermore, formaldehyde crosslinking is known to introduce artifacts (Gavrilov et al., 2015) and even DNA damage (Kawanishi et al., 2014). To address these potential issues, instead of performing the classical SSiNGLe protocol that starts with crosslinked nuclei, we isolated HMW DNA directly from K562 cells and performed SSiNGLe starting with the polyA-tailing step directly on the genomic DNA without any prior fragmentation or crosslinking (Materials and Methods). As shown in Figures 4A–C (Supplementary Table S2), we could observe the same trends as in the standard SSiNGLe protocol, thus excluding the possibility that the enrichment of the hotspots of SSBs around the TSSs is caused by MNase fragmentation or formaldehyde crosslinking.

Enrichment of hotspots around TSSs is not caused by well-characterized DNA cleavage mechanisms at promoters.

The observed enrichment of hotspots of breaks around TSSs could be explained by two well-understood mechanisms that have been previously found to have preferences for promoter regions. One of them is the activity of the apoptotic DNA fragmentation machinery that has been previously found to favor promoters of genes (Fullwood et al., 2011). While K562 cells grown under regular conditions and PBMCs extracted from healthy individuals would be expected to have low levels of apoptosis, it is hard to totally exclude some background activity of the apoptotic nucleases and/or the presence of a low fraction of cells that are undergoing cell death. Besides apoptotic DNA fragmentation, a different possible mechanism responsible for the generation of TSS-SSBs could be the TOP2 activity. Topoisomerases introduce breaks into DNA to resolve various topological issues (Pommier et al., 2016). While these breaks are transient (Pommier et al., 2016), it is conceivable that some of them could still be detected, especially because TOP2 can generate SSBs with 3′-OH termini accessible to the SSiNGLe method (Deweese and Osheroff, 2009). Furthermore, as discussed in detail below, TOP2 has been shown to associate with promoters of multiple genes in a dynamic fashion in response to transcription stimulation, and has been widely implicated in generating transcription-activating breaks. While TOP2-mediated cleavage eventually produces DSB, the enzyme has two subunits that cleave DNA independently and not at the same time (Deweese and Osheroff, 2009). Therefore, the presence of TOP2-generated SSBs at any given moment in time is expected (Deweese and Osheroff, 2009).

To test these possibilities, we observed changes in the kinetics of enrichment of the hotspots of TSS-SSBs originally found in untreated, normally grown K562 cells, in a time course of treatment with inhibitors of caspase and TOP2 activities (Figure 1A). Specifically, we employed a caspase inhibitor Z-DEVD-FMK that was shown to inhibit the enzyme activity and apoptosis in a number of studies performed either in cultured cells or *in vivo* (Clark et al., 2000; Wang et al., 2000; D'Amelio et al., 2011). In addition, we used two inhibitors of TOP2 — merbarone and ICRF-187 — because multiple studies have shown that these drugs inhibit only the catalytic activity of TOP2 without causing DNA breaks. Merbarone was shown in multiple subsequent studies to inhibit TOP2 activity without inducing DNA breaks (Drake et al., 1989; Chen and Beck, 1993; Fortune and Osheroff, 1998; Herrero-Ruiz et al., 2021). For example, in the most recent study by Herrero-Ruiz et al., merbarone treatment of human RPE-1 cells resulted in TOP2 inhibition with no detectable accumulation of neither TOP2 cleavage complexes nor DSBs (Herrero-Ruiz et al., 2021). ICRF-187 (dexrazoxane) belongs to the bisdioxopiperazine family of anticancer drugs that inhibit the catalytic activity of TOP2 at a different stage in its catalytic cycle than merbarone (Pommier et al., 2010); however, just like merbarone, ICRF-187 have been shown in multiple studies to do so without inducing free 3′-OH ends at the sites of TOP2 cleavage complexes (Ishida et al., 1991; Tanabe et al., 1991; Sehested et al., 1993). We reasoned that concordant results obtained from treating cells independently with the two types of TOP2 inhibitors that belong to very different chemical classes of molecules that can poison the enzymes at different stages of the catalytic cycle, but without inducing DNA breaks, would provide strong arguments for or against the involvement of TOP2 activity in the generation of the hotspots of TSS-SSBs.

Since the repair kinetics of the TSS-SSBs hotspots are not known, it is not clear whether short-term inhibition of the activities that generate these breaks would be sufficient to see a change in the abundance of the hotspots. In other words, if a break at a certain genomic position is long-lived due to inefficient repair, short-term inhibition of the enzyme activity that generates it may not be sufficient to see a change in the abundance of breaks at that position. Therefore, we employed a time-course strategy where K562 cells would be treated for variable lengths of times with the 3 inhibitors and their effects on the abundance of the hotspots detected in the untreated cells would be compared to the DMSO control. K562 cells are known to be resistant to apoptosis, in particular, induced by various TOP2 inhibitors (Ritke et al., 1994; Dubrez et al., 1995), due to the anti-apoptotic activity of the BCR-ABL fusion protein expressed from the Philadelphia chromosome present in these cells (Amarante-Mendes et al., 1998; Horita et al., 2000). For example, treatment of these cells with a high concentration (100 µM) of ICRF-187 resulted in visible activation of caspase-3 only after 48 h (Hasinoff et al., 2001). These results were consistent with our own findings where no induction of apoptosis was observed in K562 cells treated with another TOP2 inhibitor etoposide before 36 h of treatment (Cao et al., 2019). Therefore, we have chosen 6, 12, 24, 36 and 48 h as time points at which two biological replicas of K562 cells were treated separately with Z-DEVD-FMK, ICRF-187, merbarone, or DMSO control and then subjected to SSiNGLe profiling of SSBs. Finally, we have chosen high concentrations of merbarone and ICRF-187 (100 µM for either drug), but within range of what has been previously used in K562 cells for these drugs (Fattman et al., 1996; Hasinoff et al., 2001), to ensure that possible absence of the TOP2 effect could not be likely attributed to the inability to completely inhibit the TOP2 activity.

**FIGURE 5:**
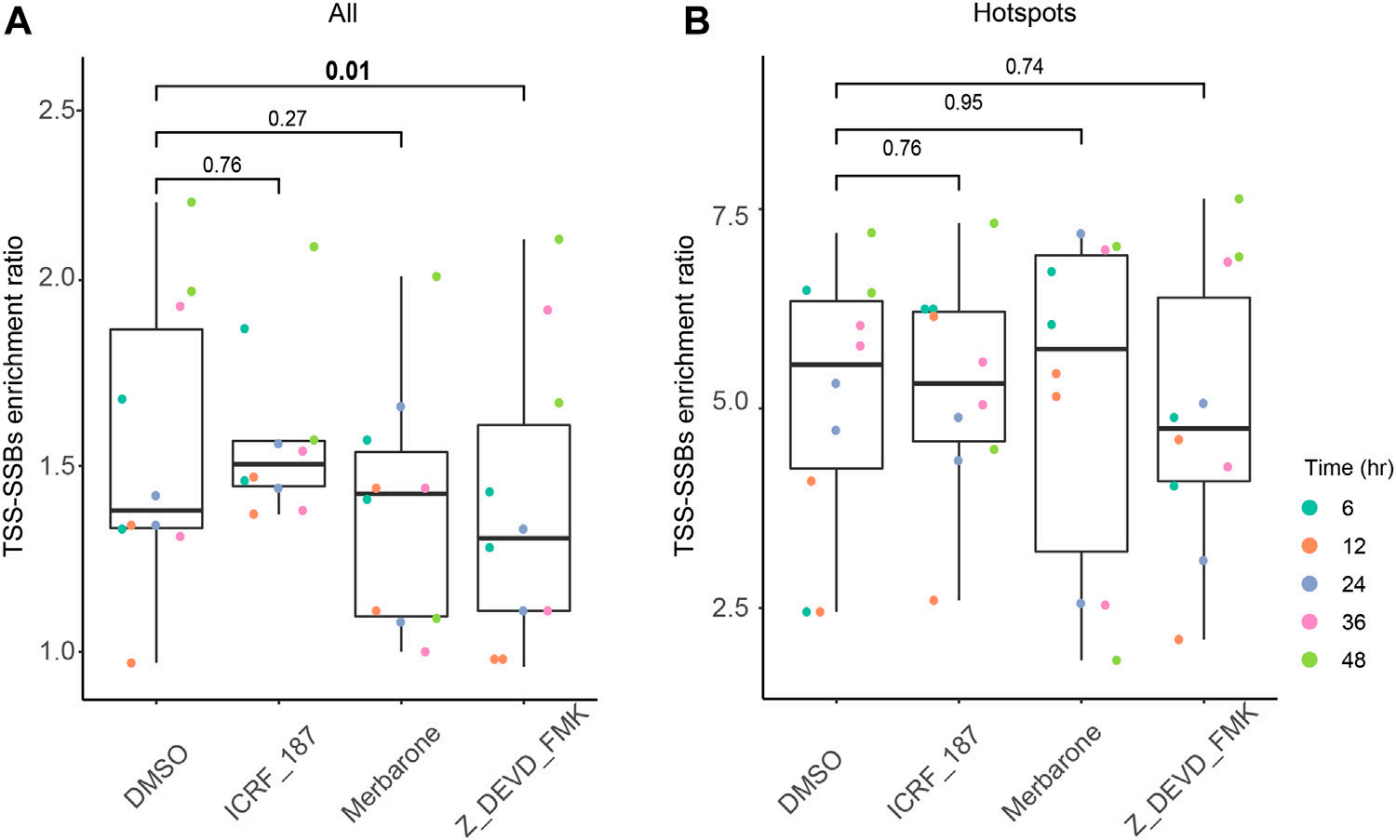
Effects of the caspase and TOP2 catalytic inhibitors on the relative enrichment of all breaks and hotspots around TSSs. The box plots of the TSS-SSBs enrichment odds ratios (Y-axes) are shown for each time point (see the inset on the right) of each treatment (X-axes) for all breaks **(A)** and hotspots **(B)**. The vertical connecting lines represent the corresponding *p*-values of the differences in the TSS-SSBs enrichment odds ratios between the various inhibitor treatments and the DMSO controls. See Supplementary Table S5 and Materials and Methods for more details.

To quantify the effects of various treatments on the enrichment of all breaks or hotspots around TSSs, we calculated the corresponding TSS-SSBs enrichment ratios (Materials and Methods) for each sample based on unique positions of all breaks detected in that sample (Figure 5A) or only positions that overlapped hotspots detected in the untreated K562 cells grown under normal conditions (Figure 5B). Therefore, any potential hotspots caused by the drug treatments would be excluded from the analyses. For both all breaks and hotspots, we combined breaks found on both the template and non-template strands since we have not observed many differences between the two strands in K562 (Figure 2F).

As illustrated in Figures 5A,B, we observed no statistically-significant differences between DMSO and either ICRF-187 or merbarone treatments for either all breaks or hotspots. These results suggested that TOP2 activity is unlikely to be a major factor in the generation of either singleton breaks or hotspots around the TSSs, consistent with the transient nature of breaks generated by TOP2. On the other hand, treatments with the caspase inhibitor did produce a small (10%), but a statistically-significant drop in the enrichment of all breaks around TSSs (Figure 5A, Supplementary Table S5). However, no statistically-significant effect of this inhibitor could be observed for the enrichment of the hotspots (Figure 5B, Supplementary Table S5). These results suggest that apoptotic DNA fragmentation machinery might indeed be partially responsible for the generation of breaks around the TSS. However, this activity does not appear to significantly influence the generation of the hotspots of breaks around the TSSs.

Moreover, we then explored the potential involvement of topoisomerase type IB (TOP1)—a type of topoisomerases that generates SSBs that has also been implicated in transcription regulation (Puc et al., 2017)—in the generation of TSS-SSBs. TOP1 enzymes generate breaks whose 3′ ends are covalently linked to the enzyme (Pommier et al., 2016) and thus should not be detected by SSiNGLe, thus making TOP1 an unlikely source of TSS-SSBs. However, the involvement of this enzyme can not be totally excluded based on this fact alone since the repair of trapped TOP1-DNA products of aborted TOP1 activity that involves tyrosyl–DNA phosphodiesterase 1 and polynucleotide kinase 3′-phosphatase generates 3′-OH termini (Caldecott, 2008; Kawale and Povirk, 2018). Therefore, we took advantage of the SSB profiles from the time course of treatment of K562 cells with a TOP1 poison SN-38 that we have previously performed using the same time points (Cao et al., 2019). If TOP1 plays a significant role in the generation of TSS-SSBs, we would expect that SN-38 would cause a change in the enrichment of these breaks relative to the DMSO control. However, we observed no statistically-significant differences between DMSO and SN-38 treatments for either all breaks or hotspots (Supplementary Table S5). Overall, these results suggest that mechanism(s) other than TOP1, TOP2 or apoptotic DNA fragmentation are likely responsible for the production of the hotspots around the TSSs.

### Association of individual breaks and hotspots with cytosines

**FIGURE 6:**
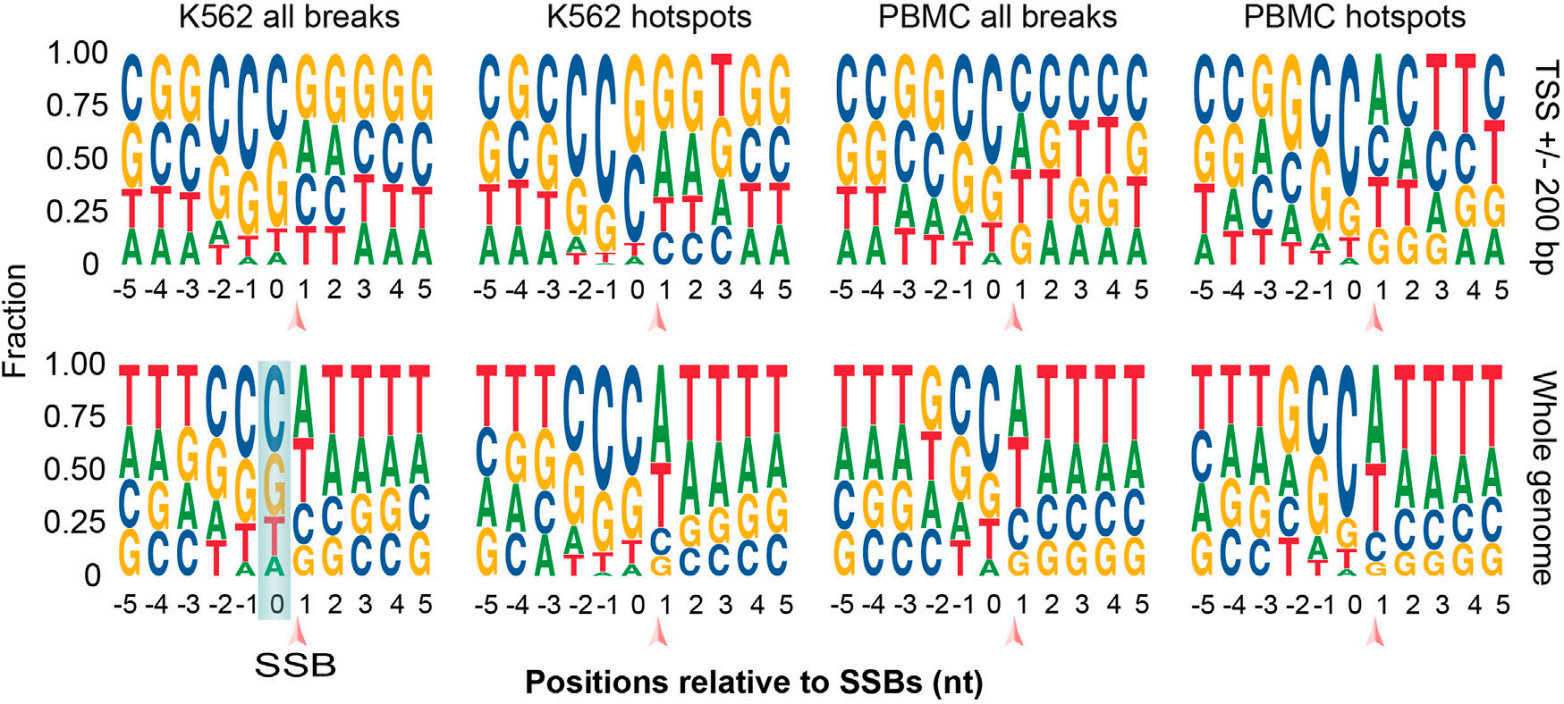
Sequence motif analysis in the immediate vicinity of all breaks and hotspots. Logo plots for the sequences around all SSBs or hotspots located within ±200 of TSSs (top) or anywhere in the genome (bottom) are shown for K562 and PBMCs. SSBs are located between the positions 0 and 1 as indicated by the arrows. See Supplementary Table S6 for more details.

To gain additional clues into the possible mechanisms of generation of TSS-SSBs, we analyzed sequence motifs in the ±5 bp windows around all SSBs and hotspots of breaks found anywhere in the genome or just in the vicinity of TSSs for both cell types. Interestingly, all breaks or hotpots found anywhere in the genome tends to occur in T-rich sequence context in both cell types, while TSS-SSBs tended to occur in a more GC-rich environment (Figure 6, Supplementary Table S6). However, strikingly, nucleotides at the positions 0 — immediately upstream of SSBs—and -1 have shown a prominent enrichment in cytosine in both PBMCs and K562 and in breaks found either around TSSs or anywhere in the genome (Figure 6, Supplementary Table S6). However, while in K562 the highest cytosine enrichment was at position -1, in PBMCs it was at position 0 (Figure 6, Supplementary Table S6). While the cytosine enrichment was prominent for all breaks and hotspots, it was higher for the latter (Figure 6, Supplementary Table S6). The second most common nucleotide at the positions -1 and 0 was guanine with adenine and thymine being significantly less abundant (Figure 6, Supplementary Table S6). Still, cytosine was significantly more frequent than guanine: except position 0 in TSS-SSBs breaks and hotspots found in K562, cytosine was the dominant base in positions -1 and 0 in all other contexts (Figure 6, Supplementary Table S6). Overall, the median C/G ratios in the positions -1 and 0 were 1.4 and 1.9 for all breaks and hotspots found anywhere in the genome across the 2 cell lines. These values increased to 1.5 and 2.1 for all breaks and hotspots around TSSs.

The preference for cytosine is potentially important because the presence of modified cytosine produced either by methylation (5-methylcytosine) or deamination (uracil) is relatively common in mammalian DNA (Nabel et al., 2012). Removal of both forms of modified cytosine by the BER pathway of DNA repair generates SSBs as intermediates of the repair process (Drohat and Coey, 2016). Thus, it is quite possible that breaks associated with 5-methylcytosine would be observed around TSSs given the prominent role this modification plays in the regulation of gene expression. However, the 5-methylcytosine typically happens in the CpG context in mammals (Nabel et al., 2012). Therefore, enrichment of guanine would be expected downstream of the breaks caused by the repair of the 5-methylcytosine. Consistent with this, some enrichment for guanine could be detected downstream of K562 breaks associated with TSSs. However, the breaks found in PBMCs did not show this trend (Figure 6, Supplementary Table S6). On the other hand, cytosine can be deaminated either spontaneously or via action of APOBEC3 family of enzymes (Nabel et al., 2012). The observed motifs were not consistent with the known sequence preferences of most APOBEC3 enzymes that are known to have strong preference for T at the -1 position (Salter et al., 2016). However, the APOBEC3G enzyme has a preference for C at position -1 (Salter et al., 2016), consistent with our findings in both cell types (Figure 6, Supplementary Table S6).

## Discussion

In this study, we found that genomic positions where SSBs preferentially occur in different cells of the same cell type exist in the human genome. Interestingly, such positions tend to be also shared by different cell types. Strikingly, the SSB hotspots tend to occur in the immediate vicinity around the TSSs, and this tendency is significantly stronger for the hotspots than for the singleton breaks. Enrichment of SSB hotspots around TSSs that is also dependent on the level of gene expression is consistent with the results of genome-wide profiling of SSBs obtained using SSB-Seq, a different method to map SSBs genome-wide (Baranello et al., 2014). However, SSB-Seq does not provide nucleotide level resolution and therefore, single-nucleotide sites of SSB hotspots could not be identified in that study (Baranello et al., 2014). Still, the mechanistic reason and biological significance behind this phenomenon are still unknown. It is possible that, for example, the repair efficiency of breaks around TSSs is slower, potentially due to the different chromatin environment around TSSs compared to elsewhere in the genome. In fact, a complex relationship between rates of repair of DSBs, their locations in active genes and cell cycle has been identified using advanced nuclear interaction mapping techniques (Aymard et al., 2017). Furthermore, DNA repair efficiencies are known to vary in a different sequence or structural contexts (Sassa and Odagiri, 2020), and it is, therefore, possible that sequences around TSSs are repaired slower by SSBR.

On the other hand, specific regions of the genome could be more prone to the formation of SSBs (and other types of DNA damage) due to unique events related to DNA metabolism, topological changes, or chromatin reprogramming happening at these regions, as recently found in neurons (Caldecott et al., 2022). Two independent studies have demonstrated that sites of DNA repair in this cell type are enriched at specific regions of the genome (Reid et al., 2021; Wu et al., 2021). Interestingly, in one study, such sites of DNA repair were specifically associated with SSBR and enriched at enhancers (Wu et al., 2021). The authors suggested increased mobility in response to transcriptional activation, higher susceptibility to DNA damage, and metabolic events associated with a high degree of epigenetic reprogramming as potential mechanisms behind the high enrichment of SSBs in these regulatory regions (Wu et al., 2021).

However, our results are also consistent with a growing realization that the effect of DNA damage on cellular physiology is more nuanced than previously thought. While DNA damage has long been thought of as a purely undesirable and deleterious effect on a cell (Hoeijmakers, 2009; Maynard et al., 2015), a number of studies (Ju et al., 2006; Perillo et al., 2008; Larsen et al., 2010; Le May et al., 2012; Bunch et al., 2015; Madabhushi et al., 2015; Puc et al., 2015; Trotter et al., 2015) have uncovered a more complex situation where specific types of DNA lesions, but most commonly DNA breaks, at promoters and enhancers are generated in response to specific stimuli and lead to activation of transcription (reviewed by (Puc et al., 2017)). An emerging theme from these studies is that a persistent DNA break first serves as a nucleation point for binding of various protein components of cellular DNA damage response that in turn leads to transcription activation via local chromatin remodeling (Ju et al., 2006; Le May et al., 2012; Trotter et al., 2015) or changes in chromatin topology (Perillo et al., 2008; Le May et al., 2012; Madabhushi et al., 2015). Interestingly, double-strand breaks (DSBs) by themselves can also initiate transcription outside of canonical promoter regions: RNA polymerase II can be directly recruited to DSBs and initiate the production of non-polyadenylated damage-induced long non-coding RNAs (dilncRNAs) and short DDRNAs (Francia et al., 2012; Michelini et al., 2017), reviewed in (Domingo-Prim et al., 2020).

The most well-characterized transcription-inducing DNA breaks at promoters or enhancers are represented by DSBs produced via the action of TOP2, cellular enzymes that generate DNA breaks to relieve torsional stress, decatenate DNA, and separate strands during transcription, replication or other nuclear processes (Pommier et al., 2016). Usually, topoisomerase-induced DNA breaks are transient, however, under some specific circumstances, for example, under certain treatments and in certain locations in the genome, they can persist for long enough to be recognized by cellular DNA damage response machinery and act as signals for transcriptional activation (Ju et al., 2006; Bunch et al., 2015; Madabhushi et al., 2015). Transcription-activating DNA breaks can also be induced by caspase-activated DNase (CAD), normally associated with DNA fragmentation during apoptosis, and have been found to be critical for myoblast differentiation when formed in the promoter of the p21 gene leading to upregulation of its expression (Larsen et al., 2010). However, our results do not support TOP2 and CAD as the major mechanisms behind the generation of TSS-associated hotspots of breaks.

In fact, other mechanisms responsible for generating transcription-activating breaks have been also reported (Perillo et al., 2008; Le May et al., 2012; Periyasamy et al., 2015). For example, at least in some biological contexts, breaks induced by the removal of the uracil produced by the enzyme-mediated deamination of cytosine at promoters can activate transcription (Periyasamy et al., 2015). Consistent with this, we found general enrichment of SSBs immediately downstream of cytosines that are more prominent in the hotspots. Even though sequences around TSSs are naturally GC-rich, random breaks would be expected to be equally enriched in cytosines and guanines. Combined with the fact that the association with cytosines could also be observed for breaks found outside of TSSs, these results raise a possibility that a mechanism that relies on cleavage at cytosines, potentially during removal of modified cytosines by DNA repair machinery, generates relatively stable breaks around TSSs and elsewhere in the genome.

One such potential mechanism could involve the removal of uracil generated by deamination, either spontaneously or mediated by APOBEC3 family of enzymes (Nabel et al., 2012). Our sequence motif analysis is somewhat consistent with the activity of APOBEC3G, but it does not prove it. Furthermore, our analysis does not exclude the removal of 5-methylcytosince by BER or spontaneous cytosine deamination as contributing factors that can explain the observed association with cytosines. Moreover, the preference for cytosines could also be caused by processes other than repair of cytosine modifications and could represent sequence preference of some other enzymatic machinery that creates breaks at these positions or slow repair of breaks around cytosines. One possible candidate is topoisomerase type IA (TOP3) which can generate SSBs with 3′-OH termini (Pommier et al., 2016) that could be detected by SSiNGLe. However, studies of the involvement of TOP3 are complicated by the absence of inhibitors that could specifically target this class of topoisomerases (Pommier, 2013). Overall, additional studies are required to determine the mechanism responsible for the observed association between the SSBs and cytosines observed in this work.

There also exists a significant amount of controversy regarding the connection between breaks and transcription activation. First, DNA breaks at promoters are also widely known to inhibit transcription (Shanbhag et al., 2010; Pankotai et al., 2012; Kakarougkas et al., 2014; Iannelli et al., 2017), reviewed in (Caron et al., 2019). Second, a recent report by Herrero-Ruiz et al. has shown that contrary to previous results, TOP2 has a negative effect on transcription activation of early response genes, and this activity is independent of DNA breaks (Herrero-Ruiz et al., 2021). Furthermore, the authors found that breaks, either SSBs or DSBs, at promoters have a negative effect on transcription (Herrero-Ruiz et al., 2021).

Therefore, additional studies are required to understand the biological function of the TSS-associated SSB hotspots. Finally, it is also important to emphasize that majority of the hotspots found in this work in either cell type mapped outside of the annotated TSSs, CAGE peaks, or promoters. Some of them could associate with TSSs of low abundant transcripts that have not been detected by the CAGE or promoter datasets used in this work. However, it is also quite likely that some hotspots are not related to transcription initiation and represent breaks consistently occurring in different cells, but are associated with or caused by some other cellular processes, or caused by exogenous damage that has a strong preference for specific locations in the genome.

## Data Availability

The datasets presented in this study can be found in online repositories. The names of the repository/repositories and accession number(s) can be found at: https://www.ncbi.nlm.nih.gov/geo/GSE190735
